# Implications of the Admixture Process in Skin Color Molecular Assessment

**DOI:** 10.1371/journal.pone.0096886

**Published:** 2014-05-08

**Authors:** Caio Cesar Silva de Cerqueira, Tábita Hünemeier, Jorge Gomez-Valdés, Virgínia Ramallo, Carla Daiana Volasko-Krause, Ana Angélica Leal Barbosa, Pedro Vargas-Pinilla, Rodrigo Ciconet Dornelles, Danaê Longo, Francisco Rothhammer, Gabriel Bedoya, Samuel Canizales-Quinteros, Victor Acuña-Alonzo, Carla Gallo, Giovanni Poletti, Rolando González-José, Francisco Mauro Salzano, Sídia Maria Callegari-Jacques, Lavínia Schuler-Faccini, Andrés Ruiz-Linares, Maria Cátira Bortolini, for CANDELA (Consortium for the Analysis of the Diversity and Evolution of Latin America)

**Affiliations:** 1 Departamento de Genética, Universidade Federal do Rio Grande do Sul, Porto Alegre, Brazil; 2 Laboratorio de Antropología Física, Departamento de Anatomía, Facultad de Medicina, Universidad Nacional Autónoma de Mexico, Mexico City, Mexico; 3 Departamento de Ciências Biológicas, Universidade Estadual do Sudoeste da Bahia, Jequié, Brazil; 4 Instituto Federal de Educação, Ciência e Tecnologia Farroupilha, Alegrete, Brazil; 5 Instituto de Alta Investigación, Universidad de Tarapacá, Facultad de Medicina, Universidad de Chile and Centro de Investigaciones del Hombre en el Desierto, Arica, Chile; 6 Universidad de Antioquia, Medellín, Colombia; 7 Unidad de Genómica de Poblaciones Aplicada a la Salud, Facultad de Química, Universidad Nacional Autónoma de México, Mexico City, Mexico; 8 Instituto Nacional de Medicina Genómica, Mexico City, Mexico; 9 Molecular Genetics Laboratory, Escuela Nacional de Antropología e Historia, Mexico City, Mexico; 10 Laboratorio de Investigación y Desarrollo, Facultad de Ciencias y Filosofía, Universidad Peruana Cayetano Heredia, Lima, Perú; 11 Centro Nacional Patagónico, CONICET, Puerto Madryn, Argentina; 12 Departamento de Estatística, Universidade Federal do Rio Grande do Sul, Porto Alegre, Brazil; 13 INAGEMP – Instituto Nacional de Genética Médica Populacional, Porto Alegre, Brazil; 14 Department of Genetics, Evolution and Environment and UCL Genetics Institute, University College London, London, United Kingdom; Instituto de Ciencia de Materiales de Madrid - Instituto de Biomedicina de Valencia, Spain

## Abstract

The understanding of the complex genotype-phenotype architecture of human pigmentation has clear implications for the evolutionary history of humans, as well as for medical and forensic practices. Although dozens of genes have previously been associated with human skin color, knowledge about this trait remains incomplete. In particular, studies focusing on populations outside the European-North American axis are rare, and, until now, admixed populations have seldom been considered. The present study was designed to help fill this gap. Our objective was to evaluate possible associations of 18 single nucleotide polymorphisms (SNPs), located within nine genes, and one pseudogene with the Melanin Index (MI) in two admixed Brazilian populations (*Gaucho*, N = 352; *Baiano*, N = 148) with different histories of geographic and ethnic colonization. Of the total sample, four markers were found to be significantly associated with skin color, but only two (*SLC24A5* rs1426654, and *SLC45A2* rs16891982) were consistently associated with MI in both samples (*Gaucho* and *Baiano*). Therefore, only these 2 SNPs should be preliminarily considered to have forensic significance because they consistently showed the association independently of the admixture level of the populations studied. We do not discard that the other two markers (*HERC2* rs1129038 and *TYR* rs1126809) might be also relevant to admixed samples, but additional studies are necessary to confirm the real importance of these markers for skin pigmentation. Finally, our study shows associations of some SNPs with MI in a modern Brazilian admixed sample, with possible applications in forensic genetics. Some classical genetic markers in Euro-North American populations are not associated with MI in our sample. Our results point out the relevance of considering population differences in selecting an appropriate set of SNPs as phenotype predictors in forensic practice.

## Introduction

In humans and other animals, pigmentation results from melanin synthesis, which occurs in melanocytes stored in vesicles known as melanosomes. Melanocytes are found in the epidermis, hair bulb, and iris, and are directly responsible for the pigmentation of these organs and structures [1], [2]. The color spectrum observed in human eyes, hair, and skin is related to the type and quantity of melanin produced, to the size, number, and pattern of packaging of melanosomes, and to the pH of the melanosomal environment [3], [4]. There are two main melanin types: eumelanin and pheomelanin, which are responsible for the black/brown and yellow/red spectra, respectively.

The human pigmentation pathway is regulated by a network controlled by many genes, as well as by environmental, mechanical, and epigenetic factors. For instance, Quillen et al. [5] identified 76 skin pigmentation candidate genes from the Online Mendelian Inheritance in Man (OMIM) database using the following key-words: “color” and “pigmentation”. This gene set included both classical and non-classical genes. Candidate pigmentation genes identified to date act over different stages of the above-mentioned processes [6]. Additional information about the function, localization, number of exons, and other relevant data related to these and others genes can be found in Cerqueira et al. [7], [8].

A considerable number of genetic studies involving normal and pathological variation in human pigmentation have emerged recently [5], [8]–[19]. Some of these investigations showed that it is possible to predict human color phenotypes from genotype data with different levels of accuracy, highlighting the importance of these types of studies for forensic practices [8], [16]. Furthermore, it has been suggested that some variants associated with pigmentation also confer susceptibility or resistance to skin cancer [12], [20], [21], since the presence of various skin tones is attributed to adaptation to diverse environments after dispersal of the anatomically modern *Homo sapiens* from Africa to other continents [9], [13]–[15], [22]–[25].

Additionally, these studies documented the extensive diversity of pigmentation genotype-phenotype architecture across human populations. For example, Quillen et al. [5] showed that in addition to the classical genes *SLC24A5* and *SLC45A2*, others, such as *OPRM1* and *EGFR*, have also contributed to differences in pigmentation between Native Americans and Europeans. Moreover, Norton et al. [6] suggested that polymorphisms in *SLC24A5, SLC45A2*, and *TYR* have a predominant role in the evolution of lighter skin color in Europeans, but not in East Asians. This indicates recent convergent evolution of lighter pigmentation phenotypes, and is suggestive the importance of natural selection in this process. Quillen and Shriver [15] evaluated skin color architecture at the population level and summarized the situation as follows: (a) despite gene flow among human populations, differences in allele frequencies for pigmentation genes are nonetheless observed; and (b) signatures of natural selection in some of these genes are observed among human populations, whereas in other genes, the signal is population-exclusive.

As noted above, several studies have indicated different populations have different genetic backgrounds for pigmentation genes. Therefore, caution should be exercised when extrapolating results from one population to another. The gene pools of contemporary admixed Brazilian populations present varying levels of contribution from continental ancestral groups (Native American, European, and African) reflected in the striking differences that have been reported among them [26], [27]. Nonetheless, few studies have examined polymorphisms in pigmentation candidate genes in these Brazilian populations to date [28]. Accordingly, the main objective of the present work was to evaluate the possible association between 18 single nucleotide polymorphisms (SNPs), located in nine candidate pigmentation genes and one pseudogene, and the Melanin Index (MI) in two Brazilian populations with different admixture levels.

## Materials and Methods

### Recruitment of study samples

This study recruited a subset of volunteers from the 1,600 Brazilian participants in the Consortium for the Analysis of the Diversity and Evolution of Latin America (CANDELA, http://www.ucl.ac.uk/silva/candela *)*. As a whole, the CANDELA project recruited 7,500 subjects over 18 years of age from five Latin America countries (Brazil, Colombia, Chile, Mexico and Peru). For this investigation, we worked with all available samples to date from persons born in Rio Grande do Sul (RS) (n = 352), the southernmost state of Brazil, and from those born in the state of Bahia (BA) (n = 148). Our goal was to evaluate possible associations between 18 SNPs and MI in two populations with contrasting ancestry formation.

The above-indicated states have distinct colonial histories: RS is characterized by more substantial European colonization (a significant number of non-Portuguese European immigrants arrived in RS in the 19^th^ century), whereas African heritage is more visible in BA with respect to both phenotypic and cultural aspects. Apart from these widely recognized characteristics, other historical, demographic, and genetic particularities can distinguish these populations [26], [27], [29]–[33].

The traditional *Gaucho* culture has roots in the South American Pampa region, whose geographical areas cover parts of RS, Argentina, and Uruguay. However, in modern Brazil, the word *Gaucho* (*Gaúcho* in Portuguese) is used to refer to anyone born in RS, and not only to those born in the Pampean region of this state. In the present study, we employ this term to refer to any volunteer from RS, whereas the term *Baiano* is used to identify individuals from BA.

This project was approved by the Research Ethics Committees of the Universidade Federal do Rio Grande do Sul, Hospital de Clínicas de Porto Alegre, and the Universidade Estadual do Sudoeste da Bahia (Resolutions 18208/2010, 100565/2011, and 212/2010, respectively). All subjects signed an informed consent form approved by the above-mentioned ethics committees, in accordance with the Declaration of Helsinki.

### MI measurements

The CANDELA protocol involved the acquisition of data on physical appearance, including a quantitative measure of skin color using the Derma spectrometer (DSM II ColorMeter, CyberDerm Inc., USA), which provides the MI. In humans, values ranging from 20 to 100 reflect fairer to darker skin tones. MI was measured on the proximal medial portion of both arms to generate a single average value. This location was chosen because it captures the constitutive skin pigmentation (the basal quantity of melanin), with potentially little effect of sun exposure [5], [34].

### Genetic analysis

DNA was extracted from total blood samples using the salting out method [35] or with the QIAamp 96 DNA blood Kit (Qiagen), and genotyping procedures were performed with KASP genotyping assay (LGC Genomics: www.lgcgenomics.com). Eighteen SNPs were investigated: rs1524668 (*ADAM17*), rs4785763 (*AFG3L1,* a pseudogene), rs6058017 (*ASIP*), rs1129038 (*HERC2*), rs1805009, rs1805008, and rs1805007 (*MC1R*), rs1800407, rs1800401, and rs1800414 (*OCA2*), rs1426654 (*SLC24A5,* also identified as *NCKX5*), rs6867641, rs26722, and rs16891982 (*SLC45A2,* also identified as *MATP*), rs3750965 and rs3829241 (*TPCN2*), and rs1042602 and rs1126809 (located at the *TYR* gene). These polymorphisms were chosen because previous studies have linked them either directly or indirectly with the human pigmentation pathway (Table S1 in File S1).

### Statistical analyses

Allele frequencies were estimated by gene counting. Agreement of genotype frequencies with Hardy-Weinberg equilibrium (HWE) was tested using the “Utility programs for analysis of genetic linkage” [36]. Allele and genotype frequency differences were compared between *Gaucho* and *Baiano* samples using Pearson χ^2^ or Fisher exact tests. Student's *t*-test or the non-parametric Wilcoxon-Mann-Whitney tests were used to compare MI, age and ancestry (European, African and Native American) between the *Gaucho* and *Baiano* samples. The assumptions of normality and homoscedasticity were tested using Kolmogorov-Smirnov and Levene's tests.

Population structure due to admixture is a well-known confounding factor in association studies. To minimize this problem, the proportion of African, European and Amerindian ancestry was estimated using the 40 Ancestry Informative Markers (AIMs) studied in all CANDELA samples. Paschou et al. [37] identified 5,000 SNPs, highly informative for worldwide continental ancestry estimation (http://www.cs.rpi.edu/~drinep/HGDPAIMS/WORLD_5000_INFAIMs.txt). Allele frequencies in Native Americans are available for a fraction of these markers [38]. The 40 markers with the highest inter-geographical group allele-frequency differences, together with the lowest intra-geographical group variation were selected for use in the CANDELA analysis. ADMIXTURE software [39] was used to estimate the proportions of African, European and Native American ancestry, as well as their standard errors. More details about these AIMs and the admixture analysis can be found in Ruiz-Linares et al. [40]. We also estimated the correlation between 1/MI and ancestry proportion using Pearson's product–moment coefficient, to evaluate the influence of ancestry in our dataset.

ANOVA (General Linear Models) were employed to test for the association of genetic polymorphisms with the melanin index (MI) by comparing these values across genotypes; Sidák-corrected multiple comparisons were performed when appropriate. The proportions of African and European ancestries were included in the model as covariates. Note that covariates should be included in the ANOVA only if they are associated with both dependent (melanin index) and independent (genotype) variables. We also included the group label (*Gaucho* vs. *Baiano*) as an additional covariate in the total sample analysis to assess whether the results were robust to this additional known level of population structure. A Bonferroni-corrected *p* value threshold of 0.0028 was considered statistically significant for ANOVA tests.

The statistical analyses were performed using WinPEPI version 4.0 [41], or PASW (former SPSS) statistical package, version 18.0.

## Results

### Management of quantitative data and gene frequency analyses

Since the assumptions for the ANOVA and t-tests were not satisfied for age and MI, we used 1/MI and 1/age transformations. Non-parametric methods were used when this transformation was not enough to satisfy those assumptions. The mean age for the total sample (N = 500) was 25 (range 18–85) years, 168 (33.6%) being males and 332 (66.4%) females. The *Gaucho* mean age (26; range 18–85) years differed significantly from the *Baiano* mean age (23; 18–62) (*p*<0.001). The mean MI of the total sample was 32.90 (range 22.04–76.40), while *Gaucho* and *Baiano* showed average MI values of 31.54 (22.04–76.40) and 36.68 (23.72–65.01), respectively. These results indicate that the *Baiano* group presents a darker skin color compared with the *Gaucho* group (*p*<0.001).

In the *Gaucho* sample, genotype frequencies were in accordance with those expected by HWE, except for *SLC24A5* rs1426654 (χ^2^ = 11.051, *p*<0.01), which showed a lower number of heterozygotes than expected. For the *Baiano* group, only the genotype frequencies of *SLC45A2* rs16891982 were not in HWE (χ^2^ = 6.911, *p*<0.01), also due to a lower than expected number of heterozygotes. Allele and genotype frequencies were statistically different between the 2 populations for *ASIP* rs6058017, *HERC2* rs1129038, *SLC24A5* rs1426654, and *SLC45A2* rs16891982 (Table S2 in File S1).

### Association between MI and genotypes

Aiming to evaluate possible population stratification due to admixture, we first tested the differences between *Gaucho* and *Baiano* samples with respect to European, African, and Native American ancestries measured using the 40 Ancestry Informative Markers employed in the CANDELA Consortium. The two groups differed for African and European contributions (*p*<0.001), as well as Native American ancestry (*p* = 0.017). The percentage mean values are: *Gaucho*: European, 84.86; African, 6.99; and Native American, 8.15; *Baiano*: 61.47, 29.91, and 8.62, respectively (Table S3 in File S1).

We also observed a moderate correlation between 1/MI and European (r = 0.516; *p*<0.001); and African (r = –0.598; *p*<0.001) proportions; and a weak correlation between 1/MI and Native-American ancestry (r = –0.167; *p*<0.001). However, only the African and European ancestries were associated with both genotypes and the melanin index, as opposed to the Native American ancestry. Sex was not associated with MI (*p* = 0.817) and a negligible correlation (–0.089; *p* = 0.046) was observed between MI and age. Therefore, in the ANOVA analysis of the association between the 18 genetic markers and MI, we used European and African proportions as covariates to adjust for population stratification.

Three sets of ANOVA analyses were performed to evaluate the association between MI and genotypes for the 18 markers: for the total sample (N = 500), for the *Gaucho* subsample (N = 352), and for the *Baiano* subsample (N = 148). The results are presented in Table 1. In the total sample, four polymorphisms showed a statistically significant association with MI: *SLC24A5* rs1426654 (*p*<0.001) and *SLC45A2* rs16891982 (*p*<0.001); and *HERC2* rs1129038 (*p* = 0.001), and *TYR* rs1126809 (*p* = 0.001). But when the *Gaucho* and *Baiano* populations were considered separately, only two of these SNPs (*SLC24A5* rs1426654 and *SLC45A2* rs16891982) remained significantly associated with MI (*p*<0.001). The inclusion of the group label (*Gaucho* vs. *Baiano*) as an additional covariate in the combined population analysis did not change our results (data not shown).

**Table 1:** Ancestry adjusted ANOVA tests for association between Melanin Index (MI)^a^ and genotypes in two admixed Brazilian populations.

		Total sample (N = 500)		*Gaucho* (N = 352)		*Baiano* (N = 148)	
Gene	SNP	F	*p*	F	*p*	F	*p*
*ADAM17*	rs1524668	0.578	0.562	0.817	0.443	0.331	0.719
*AFG3L1*	rs4785763	0.167	0.846	0.116	0.891	0.090	0.914
*ASIP*	rs6058017	0.273	0.761	0.131	0.877	0.462	0.631
*HERC2*	rs1129038	**7.599**	**0.001**	3.418	0.035	3.154	0.046
*MC1R*	rs1805009	4.436	0.036	6.698	0.010	0.096	0.758
*MC1R*	rs1805008	0.299	0.585	0.315	0.575	b	b
*MC1R*	rs1805007	0.267	0.605	0.440	0.507	0.498	0.482
*OCA2*	rs1800407	8.195	0.004	3.656	0.057	6.073	0.015
*OCA2*	rs1800401	0.262	0.770	0.803	0.371	0.018	0.982
*OCA2*	rs1800414	0.863	0.353	0.892	0.346	0.036	0.850
*SLC24A5*	rs1426654	**30.816**	**<0.001**	**21.946**	**<0.001**	**10.441**	**<0.001**
*SLC45A2*	rs6867641	0.400	0.671	0.444	0.642	9.795	0.089
*SLC45A2*	rs26722	2.445	0.088	3.485	0.032	0.595	0.553
*SLC45A2*	rs16891982	**57.755**	**<0.001**	**32.974**	**<0.001**	**26.747**	**<0.001**
*TPCN2*	rs3750965	0.642	0.527	0.814	0.444	0.079	0.924
*TPCN2*	rs3829241	0.482	0.618	1.018	0.363	0.273	0.761
*TYR*	rs1042602	3.637	0.027	1.196	0.304	4.974	0.008
*TYR*	rs1126809	**6.905**	**0.001**	3.853	0.022	3.874	0.023

a ANOVA tests performed on 1/MI.

b Not tested due to scarcity of data (only n = 4 individuals with genotype CC).

The average values observed for MI suggest that for loci *HERC2* rs1129038, *SLC45A2* rs16891982 and *TYR* rs1126809, the respective alleles A, C, and A, present a dominant effect (Table 2), while alleles A and G of the *SLC24A5* rs1426654 locus seem to have a codominant effect. Alleles *HERC2* rs1129038 G, *SLC45A2* rs16891982 C, *SLC24A5* rs1426654 G, and *TYR* rs1126809 G were associated with darker skin pigmentation (Table 2).

**Table 2:** MI crude and ancestry-adjusted means, according to the genotypes of four polymorphisms for which an association with the Melanin Index (MI) was observed.

Polymorphisms and genotype	Total sample			Gaucho			Baiano		
	N	MI mean	Adj MI mean^a^	N	MI mean	Adj MI mean^a^	N	MI mean	Adj MI mean^a^
HERC2 rs1129038									
GG	157	35.2	34.4 R	65	N/A	N/A	92	N/A	N/A
GA	140	32.8	32.6 S	94	N/A	N/A	46	N/A	N/A
AA	43	30.8	31.8 S	40	N/A	N/A	3	N/A	N/A
									
GG	23	49.8	38.7 R	9	49.6	41.0 R	14	49.9	41.7 R
GA	104	37.3	35.3 S	51	34.9	34.0 S	53	39.6	38.5 R
AA	366	32.1	32.0 T	288	31.2	30.9 T	78	35.4	34.9 S
SLC45A2 rs16891982									
CC	66	41.3	36.1 R	24	39.7	35.4 R	42	42.2	39.0 R
CG	164	36.1	34.9 R	110	33.8	33.3 R	54	41.0	39.4 R
GG	252	30.7	31.0 S	209	30.5	30.3 S	43	31.7	32.3 S
TYR rs1126809									
GG	338	34.8	33.4 R	229	N/A	N/A	109	N/A	N/A
GA	132	32.6	32.2 S	98	N/A	N/A	34	N/A	N/A
AA	18	30.0	30.5 S	16	N/A	N/A	2	N/A	N/A

N/A  =  No association detected;

a Means indicated by the same letter do not differ at the 0.05 level (Sidák-corrected multiple comparisons based on 1/MI).

## Discussion

Knowledge about the connection between genetic variation and complex traits has improved markedly in recent years. Nevertheless, current knowledge of genetic predictors of skin color remains limited [42], particularly with respect to populations outside of the European-North American axis. Our intention was to help fill this gap by demonstrating the effect of genetic variants for pigmentation in two admixed Brazilian populations.

Our results showed that just two polymorphisms (*SLC24A5* rs1426654 and *SLC45A2* rs16891982) seem to behave relatively consistently as skin color predictors in admixed samples. *HERC2* rs1129038 and *TYR* rs1126809 were significantly associated with MI in the total sample analyses only. We do not discard the possibility that these two markers might also be relevant, but additional studies are necessary to confirm their real importance for skin pigmentation. It is possible that their association in *Gaucho* and *Baiano* disappeared due to small sample size when these populations were considered separately. On the other hand, some classical markers associated with skin pigmentation in Europeans and others populations (Table S1 in File S1) are not associated in our admixed samples (for example, *ASIP* rs6058017, *MC1R* rs1805009, *OCA2* rs1800414, *SLC45A2* rs6867641), indicating the possibility that they are population-specific. Therefore, they should be avoided for general inferences in forensic DNA phenotyping. This notable absence of association between classical skin pigmentation loci (including *ASIP* and *MC1R*) and skin color also was noted by Beleza et al. [43] studying Cape Verdean subjects with extensive West African/European admixture. On the other hand, similarly to us, they found a significant association of *SLC24A5* rs1426654 and *SLC45A2* rs16891982 with this trait.

Pigmentation differs due to the amount and type of melanin synthesized in melanocytes, and to melanosome shape and distribution [3]. Complex gene and protein networks are related to these processes, and it is likely that their architectures differ, at least in part, across diverse human populations. Our findings have important forensic implications, since some authors have suggested that eye and hair color predictions can be developed independently of the biogeographic ancestry of the population investigated [17], [18], [44], [45]. Results of the present study indicated that for skin color this assumption appears to be only partially true.

Despite the fact that we observed an intermediate effect of heterozygote genotypes in the majority of significant SNPs, it is clear that heterozygotes for some polymorphisms had MI means similar to those of one of the homozygotes. The description of the specific effect of each genotype is therefore extremely important. For instance, *HERC2* rs1129038 and *TYR* rs1126809 suggest a dominant relationship between alleles, as found by Cook et al. [46] in a study of the expression of pigmentation genes across different genotype profiles. SNPs S*LC45A2* rs16891982 and *SLC24A5* rs1426654 were previously associated with skin color in European and/or Asian populations [6], [17], [18], [46]–[48], and our results indicate similar association in admixed populations. On the other hand, *HERC2* rs1129038 was previously associated with eye and hair color only [49], [50]. This polymorphism is in high linkage disequilibrium with *HERC2* rs12913832 [50], [51], which, in turn, is associated with eye, hair, and skin color [46], [50], and seems to have an on-off effect on the *OCA2* gene function [52], [53]. Our study shows, for the first time, that *HERC2* rs1129038 can have a role in skin color, at least in some populations, such as those investigated here (Total sample; Table 1).

Interestingly, some studies have suggested that the *SLC45A2* rs16891982 C allele was associated with darker skin and/or brown eyes, whereas in other investigations, this allele was associated with lighter skin and/or blue eyes (Table S1 in File S1). This discrepancy may be due to the fact that C/G differences can easily lead to reading mistakes. Our data indicated that the C allele (Phe) was related to higher MI values, thus corroborating the propositions of Norton et al. [6], Spichenok et al. [17], and Walsh et al., [18].

In general, the effects described here for the SNPs statistically associated with skin color are in agreement with their functionality, as described in the literature. For instance, Ginger et al. [54] demonstrated that the expression of the *SLC24A5* gene product is required for the production of melanin in differentiating human epidermal melanoblasts. Also, a functional study conducted by Sturm [55] found that in SLC24A5*111Ala homozygotes the amount of melanin in cultured melanocytes increased by 2.2 times, as compared to *Thr111Thr*. More information about the biological effects of other SNPs studied here in human pigmentation may be viewed in Table S1 in File S1.

## Conclusion

The main implication of our study is the establishment of some genetic markers as skin color predictors in admixed populations. Two SNPs (*SLC24A5* rs1426654 and *SLC45A2* rs16891982) were significantly associated with MI in all analyses. Two other markers showed suggestive results (*HERC2* rs1129038 and *TYR* rs1126809), indicating the need for additional studies for confirmation. An interesting finding is that while some polymorphisms seem to behave consistently as skin color predictors, others seem to be population-specific, indicating that genotype-phenotype relationships found in one population cannot be uncritically extrapolated to another.

## Supporting Information

File S1 Table S1, Characteristics of the SNPs studied. Table S2, Genotype and allele frequencies for 18 SNPs in the *Gaucho* (N = 352) and *Baiano* (N = 148) samples. Table S3, Mean and range values for the ancestry proportion (%) in the *Baiano* and *Gaucho* samples.
(DOCX)
