# Correction: Implications of the Admixture Process in Skin Color Molecular Assessment

**DOI:** 10.1371/journal.pone.0109451

**Published:** 2014-09-24

**Authors:** The PLOS ONE Staff

There is an error in the last sentence of the penultimate paragraph of the Discussion. The sentence should read:

Our data indicated that the C allele (corresponding to G allele in mRNA or Leucine in transcript) was related to higher MI values, thus corroborating the propositions of Norton et al. [6], Spichenok et al. [17], and Walsh et al., [18].
