# Supplementary material for: Implications of the Admixture Process in Skin Color Molecular Assessment
Source: PLoS One. 2014 May 8;9(5):e96886. doi: 10.1371/journal.pone.0096886 (PMC4014568; doi:10.1371/journal.pone.0096886)
Supplement: File S1 — Table S1, Characteristics of the SNPs studied. Table S2, Genotype and allele frequencies for 18 SNPs in the Gaucho (N = 352) and Baiano (N = 148) samples. Table S3, Mean and range values for the ancestry proportion (%) in the Baiano and Gaucho samples. (DOCX) [file pone.0096886.s001.docx]

Table S1. Characteristics of the SNPs studied^a^.

| **Gene** | **SNP ID** | **Localization** | **Number of exons** | **Mutation** | **Probable allele function** | **Studied populations** | **Reference (s)** |
| --- | --- | --- | --- | --- | --- | --- | --- |
| *ADAM17*^b^ | rs1524668 | 2p25.1 | 19 | -968C>A - 5'UTR | - | Admixed Brazilian populations | Present study |
| *AFG3L1* | rs4785763 | 16q24.3 | pseudogene | A>C | A = red hair, freckles/ C = not-red hair, no freckles | Europeans | [1] |
| *ASIP* | rs6058017 | 20q11.22 | 4 | 8818G>A - 3'UTR | G = more melanin, no freckles/ A= less melanin, freckles | African-Americans from Washington, Europeans, East Asians | [1-4] |
| *HERC2* | rs1129038 | 15q13.1 | 93 | G>A - 3'UTR | A = blue eyes/ G = not-blue eyes | Europeans | [5, 6] |
| *MC1R* | rs1805009 | 16q24.3 | 1 | Asp294His - G>C | G = not-red hair, not-fairer skin/ C= red hair, fairer skin | Europeans, Caucasians | [7-9] |
|  | rs1805008 |  |  | Arg160Trp - C>T | T = blonde or red hair, freckles, skin sensitivity to sun, fairer skin/ C = not-blonde or not-red hair, no freckles | Europeans, Caucasians | [1,8-10] |
|  | rs1805007 |  |  | Arg151Cys - C>T | T = blonde or red hair, freckles, skin sensitivity to sun, fairer skin/ C = not-blonde or not-red hair, no freckles | Europeans, Caucasians | [1, 8-10] |
| *OCA2* | rs1800407 | 15q13.1 | 24 | Arg419Gln - G>A | G = not-green or brown or black eyes/ A = green or blue eyes | Caucasians of Pennsylvania, Europeans | [11, 12] |
|  | rs1800401 |  |  | Arg305Trp - C>T | C = blue eyes, lighter pigmentation/ T = brown/black eyes and darker pigmentation | Caucasians of Pennsylvania | [11] |
|  | rs1800414 |  |  | His615Arg - A>G | A = darker skin/ G = fairer skin | East Asian ancestry living in Canada | [13] |
| *SLC24A5 (NCKX5)* | rs1426654 | 15q21.1 | 9 | Ala111Thr - G>A | G = more melanin/ A = less melanin | Europeans, Mixed populations, African–Americans, Asians | [4, 14, 15] |
| *SLC45A2 (MATP)* | rs6867641 | 5p13.3 | 7 | -1169C>T - 5'UTR | C = fairer skin/ T = darker skin | Caucasians, Asians, African Americans, Australian Aborigines, Spanish Basques | [16] |
|  | rs26722 |  |  | Glu272Lys - G>A | G = fairer skin, lighter brown hair, not-brown eyes/ A = darker skin, hair, and eye color | Caucasians, Asians, African-Americans, Australian Aborigines | [17] |
|  | rs16891982 |  |  | Phe374Leu - C>G | C = darker skin, brown or black eyes/ G = blue eyes, lighter or medium skin, darker skin, hair, and eye color | Caucasians, Asians, African-Americans, Australian Aborigines, Europeans, Mixed populations | [4, 12, 14, 15, 17] |
| *TPCN2* | rs3750965 | 11q13.2 | 25 | Lys376Arg - A>G | A = blonde hair / G = brown hair | Europeans | [1] |
|  | rs3829241 |  |  | Gly734Glu - G>A | A= blond hair, skin sensitivity to sun/ G= brown hair, darker skin | Europeans | [1] |
| *TYR* | rs1042602 | 11q14.3 | 5 | Ser192Tyr - C>A | C = skin sensitivity to sun, freckles, brown hair/ A = no freckles, blonde hair | Europeans | [1, 10] |
|  | rs1126809 |  |  | Arg402Gln - G>A | G= not-blue eyes, brown hair/ A=blue eyes, blonde hair, skin sensitivity to sun | Europeans | [1] |

^a^Key to abbreviations: A = Adenine, *ADAM17* = a disintegrin and metallopeptidase domain 17, *AFG3L1* = ATPase family gene 3-like 1 (pseudogene), Ala = alanine, *ASIP* = agouti signalling protein, Arg = arginine, Asp = aspartic acid, C = Cytosine, G = Guanine, Gln = glutamine, Glu = glutamic acid, Gly = glycine, *HERC2* = HECT and RLD domain containing E3 ubiquitin protein ligase 2, His = histidine, Leu = leucine, Lys = lysine, *MATP* = membrane associated transporter protein, *MC1R* = melanocortin 1 receptor, *NCKX5*= Na/Ca/K exchanger 5, *OCA2* = oculocutaneous albinism II, Phe = phenylalanine, Ser = serine, *SLC24A5* = solute carrier family 24, member 5, *SLC45A2* = solute carrier family 45 member 2, T= Thymine, Thr = threonine, *TPCN2* = two pore segment channel 2, Trp = tryptophan, *TYR* = tyrosinase (gene), Tyr = tyrosine (amino acid), UTR = untranslated region.

^b^*ADAM17* was mentioned by Norton et al. [4] as an ‘a priori pigmentation candidate gene’, with a positive selection signal in East-Asians. We selected the *ADAM17*rs1524668 SNP because the derived allele is in high frequency in Amerindians (HGDP-CEPH, http://www.cephb.fr/en/hgdp/diversity.php/).

Table S2. Genotype and allele frequencies for 18 SNPs in the *Gaucho* (N = 352) and *Baiano* (N = 148) samples**.**

|  | rs1042602 | | rs1126809 | | rs6867641 | | rs26722 | | rs16891982 | | rs1800407 | |
| --- | --- | --- | --- | --- | --- | --- | --- | --- | --- | --- | --- | --- |
|  | 1 = C/2 = A | | 1 = G/2 =A | | 1 = C/2 = T | | 1 = C/2 = T | | 1 = C/2 = G | | 1 = G/2 = A | |
| Genotypes/alleles | Gaucho | Baiano | Gaucho | Baiano | Gaucho | Baiano | Gaucho | Baiano | Gaucho | Baiano ^a^ | Gaucho | Baiano |
| 11 | 0.367 | 0.483 | 0.667 | 0.752 | 0.484 | 0.000 | 0.826 | 0.815 | **0.070** | **0.302** | 0.880 | 0.875 |
| 12 | 0.460 | 0.392 | 0.286 | 0.234 | 0.410 | 0.750 | 0.171 | 0.178 | **0.321** | **0.388** | 0.120 | 0.125 |
| 22 | 0.173 | 0.126 | 0.047 | 0.014 | 0.106 | 0.250 | 0.003 | 0.007 | **0.609** | **0.309** | 0.000 | 0.000 |
| 1 | 0.597 | 0.678 | 0.810 | 0.869 | 0.689 | 0.375 | 0.911 | 0.904 | **0.230** | **0.496** | 0.940 | 0.938 |
| 2 | 0.403 | 0.322 | 0.190 | 0.131 | 0.311 | 0.625 | 0.089 | 0.096 | **0.770** | **0.504** | 0.060 | 0.062 |
|  | rs1800401 | | rs1800414 | | rs1129038 | | rs6058017 | | rs3750965 | | rs3829241 | |
|  | 1 = C/2 = T | | 1 = A/2 = G | | 1 = G/2 = A | | 1 = G/2 = A | | 1 = A/2 = G | | 1 = G/2 = A | |
| Genotypes/alleles | Gaucho | Baiano | Gaucho | Baiano | Gaucho | Baiano | Gaucho | Baiano | Gaucho | Baiano | Gaucho | Baiano |
| 11 | 0.883 | 0.799 | 0.994 | 0.993 | **0.327** | **0.653** | **0.012** | **0.095** | 0.437 | 0.514 | 0.487 | 0.587 |
| 12 | 0.117 | 0.188 | 0.006 | 0.007 | **0.472** | **0.326** | **0.228** | **0.361** | 0.452 | 0.363 | 0.398 | 0.371 |
| 22 | 0.000 | 0.013 | 0.000 | 0.000 | **0.201** | **0.021** | **0.760** | **0.544** | 0.111 | 0.123 | 0.115 | 0.042 |
| 1 | 0.941 | 0.893 | 0.997 | 0.997 | **0.563** | **0.816** | **0.126** | **0.276** | 0.663 | 0.695 | 0.686 | 0.773 |
| 2 | 0.059 | 0.107 | 0.003 | 0.003 | **0.437** | **0.184** | **0.874** | **0.724** | 0.337 | 0.305 | 0.314 | 0.227 |
|  | rs1426654 | | rs1524668 | | rs1805009 | | rs1805008 | | rs1805007 | | rs4785763 | |
|  | 1 = G/2 = A | | 1 = G/2 = T | | 1 = G/2 = C | | 1 = C/2 = T | | 1 = C/2 = T | | 1 = A/2 = C | |
| Genotypes/alleles | Gaucho ^a^ | Baiano | Gaucho | Baiano | Gaucho | Baiano | Gaucho | Baiano | Gaucho | Baiano | Gaucho | Baiano |
| 11 | **0.026** | **0.097** | 0.407 | 0.432 | 0.971 | 0.986 | 0.914 | 1.000 | 0.945 | 0.993 | 0.045 | 0.056 |
| 12 | **0.147** | **0.366** | 0.462 | 0.480 | 0.029 | 0.014 | 0.086 | 0.000 | 0.055 | 0.007 | 0.364 | 0.347 |
| 22 | **0.827** | **0.538** | 0.131 | 0.088 | 0.000 | 0.000 | 0.000 | 0.000 | 0.000 | 0.000 | 0.591 | 0.597 |
| 1 | **0.100** | **0.279** | 0.638 | 0.672 | 0.986 | 0.993 | 0.957 | 1.000 | 0.973 | 0.997 | 0.227 | 0.229 |
| 2 | **0.900** | **0.721** | 0.362 | 0.328 | 0.014 | 0.007 | 0.043 | 0.000 | 0.027 | 0.003 | 0.773 | 0.771 |

^a^Genotype frequencies deviate from Hardy-Weinberg Equilibrium;

Allele and genotype frequencies which differ (*p* < 0.001) between *Gaucho* and *Baiano* are in bold.

Table S3. Mean and range values for the ancestry proportion (%) in the *Baiano* and *Gaucho* samples.

|  | ***Baiano* subsample** | | |  | ***Gaucho* subsample** | | |  |
| --- | --- | --- | --- | --- | --- | --- | --- | --- |
|  | Mean (SD) | Mininum | Maximum |  | Mean (SD) | Mininum | Maximum | *p* |
| European ancestry | 61.47 (22.51) | 2.42 | 100 |  | 84.86 (18.06) | 0 | 100 | < 0.001 |
| African ancestry | 29.91 (20.30) | 0 | 88.49 |  | 6.99 (10.49) | 0 | 91.13 | <0.001 |
| Native American ancestry | 8.62 (6.91) | 0 | 36.80 |  | 8.15 (9.07) | 0 | 55.99 | 0.017 |

**Supporting Information References**

[1] Sulem P, Gudbjartsson DF, Stacey SN, Helgason A, Rafnar T, et al. (2008) Two newly identified genetic determinants of pigmentation in Europeans. Nat Genet 40: 835–837.

[2] Kanetsky PA, Swoyer J, Panossian S, Holmes R, Guerry D, et al. (2002) A polymorphism in the agouti signaling protein gene is associated with human pigmentation. Am J Hum Genet 70: 770–775.

[3] Bonilla C, Boxill LA, Donald SA, Williams T, Sylvester N, et al. (2005) The 8818G allele of the agouti signaling protein (ASIP) gene is ancestral and is associated with darker skin color in African Americans. Hum Genet 116: 402–406.

[4] Norton HL, Kittles RA, Parra E, McKeigue P, Mao X, et al. (2007) Genetic evidence for the convergent evolution of light skin in Europeans and East Asians. Mol Biol Evol 24: 710–722.

[5] Eiberg H, Troelsen J, Nielsen M, Mikkelsen A, Mengel-From J, et al. (2008) Blue eye color in humans may be caused by a perfectly associated founder mutation in a regulatory element located within the HERC2 gene inhibiting OCA2 expression. Hum Genet 123: 177–187.

[6] Sturm RA, Duffy DL, Zhao ZZ, Leite FP, Stark MS, et al. (2008) A single SNP in an evolutionary conserved region within intron 86 of the HERC2 gene determines human blue-brown eye color. Am J Hum Genet 82: 424–431.

[7] [Smith R,](http://www.ncbi.nlm.nih.gov/pubmed?term=Smith%20R%5BAuthor%5D&cauthor=true&cauthor_uid=9665397) [Healy E,](http://www.ncbi.nlm.nih.gov/pubmed?term=Healy%20E%5BAuthor%5D&cauthor=true&cauthor_uid=9665397) [Siddiqui S,](http://www.ncbi.nlm.nih.gov/pubmed?term=Siddiqui%20S%5BAuthor%5D&cauthor=true&cauthor_uid=9665397) [Flanagan N,](http://www.ncbi.nlm.nih.gov/pubmed?term=Flanagan%20N%5BAuthor%5D&cauthor=true&cauthor_uid=9665397) [Steijlen PM,](http://www.ncbi.nlm.nih.gov/pubmed?term=Steijlen%20PM%5BAuthor%5D&cauthor=true&cauthor_uid=9665397) [et al.](http://www.ncbi.nlm.nih.gov/pubmed?term=Rosdahl%20I%5BAuthor%5D&cauthor=true&cauthor_uid=9665397) [(1998)](http://www.ncbi.nlm.nih.gov/pubmed?term=Rees%20JL%5BAuthor%5D&cauthor=true&cauthor_uid=9665397) Melanocortin 1 receptor variants in an Irish population. [J Invest Dermatol](http://www.ncbi.nlm.nih.gov/pubmed/9665397) 111: 119–122.

[8] Han J, Kraft P, Colditz GA, Wong J, Hunter DJ. (2006) Melanocortin 1 receptor variants and skin cancer risk. Int J Cancer 119: 1976–1984.

[9] [Latreille J,](http://www.ncbi.nlm.nih.gov/pubmed?term=Latreille%20J%5BAuthor%5D&cauthor=true&cauthor_uid=19656326) [Ezzedine K,](http://www.ncbi.nlm.nih.gov/pubmed?term=Ezzedine%20K%5BAuthor%5D&cauthor=true&cauthor_uid=19656326) [Elfakir A,](http://www.ncbi.nlm.nih.gov/pubmed?term=Elfakir%20A%5BAuthor%5D&cauthor=true&cauthor_uid=19656326) [Ambroisine L,](http://www.ncbi.nlm.nih.gov/pubmed?term=Ambroisine%20L%5BAuthor%5D&cauthor=true&cauthor_uid=19656326) [Gardinier S,](http://www.ncbi.nlm.nih.gov/pubmed?term=Gardinier%20S%5BAuthor%5D&cauthor=true&cauthor_uid=19656326) [et al.](http://www.ncbi.nlm.nih.gov/pubmed?term=Galan%20P%5BAuthor%5D&cauthor=true&cauthor_uid=19656326) [(2009)](http://www.ncbi.nlm.nih.gov/pubmed?term=Guinot%20C%5BAuthor%5D&cauthor=true&cauthor_uid=19656326) MC1R gene polymorphism affects skin color and phenotypic features related to sun sensitivity in a population of French adult women. [Photochem Photobiol](http://www.ncbi.nlm.nih.gov/pubmed/19656326) 85: 1451–1458.

[10] Sulem P, Gudbjartsson DF, Stacey SN, Helgason A, Rafnar T, et al. (2007) Genetic determinants of hair, eye and skin pigmentation in Europeans. Nat Genet 39: 1443–1452.

[11] Rebbeck TR, Kanetsky PA, Walker AH, Holmes R, Halpern AC, et al. (2002) P gene as an inherited biomarker of human eye color. Cancer Epid. Biomarkers Prev 11: 782–784.

[12] Walsh S, Liu F, Ballantyne KN, Van Oven M, Lao O, et al. (2011) IrisPlex: A sensitive DNA tool for accurate prediction of blue and brown eye colour in the absence of ancestry information. Forensic Sci Int Genet 5: 170–180.

[13] Edwards M, Bigham A, Tan J, Li S, Gozdzik A, et al. (2010) Association of the OCA2 polymorphism His615Arg with melanin content in east Asian populations: Further evidence of convergent evolution of skin pigmentation. PLoS Genet 6: e1000867.

[14] Cook AL, Chen W, Thurber AE, Smit DJ, Smith AG, et al. (2009) Analysis of cultured human melanocytes based on polymorphisms within the SLC45A2/MATP, SLC24A5/NCKX5, and OCA2/P loci. J Invest Dermatol 129: 392–405.

[15] Spichenok O, Budimlija ZM, Mitchell AA, Jenny A, Kovacevic L, et al. (2011) Prediction of eye and skin color in diverse populations using seven SNPs. Forensic Sci Int Genet 5: 472–478.

[16] Graf J, Voisey J, Hughes I, Van Daal A. (2007) Promoter polymorphisms in the MATP (SLC45A2) gene are associated with normal human skin color variation. Hum Mut 28: 710–717.

[17] Graf J, Hodgson R, Van Daal A. (2005) Single nucleotide polymorphisms in the MATP gene are associated with normal human pigmentation variation. Hum Mut 25: 278–284.
